# Depression Among Adolescents in a Rural Area of Haryana, India: A Community-Based Study Using Patient Health Questionnaire-9

**DOI:** 10.7759/cureus.18388

**Published:** 2021-09-29

**Authors:** Aditi Mohta, Sumit Malhotra, Sanjeev K Gupta, Kalaivani Mani, Bichitra N Patra, Baridalyne Nongkynrih

**Affiliations:** 1 Centre for Community Medicine, All India Institute of Medical Sciences, New Delhi, IND; 2 Department of Biostatistics, All India Institute of Medical Sciences, New Delhi, IND; 3 Department of Psychiatry, All India Institute of Medical Sciences, New Delhi, IND

**Keywords:** brain awareness, adolescent, depression, rural, community

## Abstract

Introduction: The estimates of prevalence of depression among adolescents in the Indian community are limited; most studies are institution-based. Early identification and management of depression can provide significant health dividends to the affected adolescents, and better health consequences in their adulthood.

Objectives: To determine the prevalence of depression among 10- to 19-year-old residents in a rural area of Haryana, India, and to assess factors associated with adolescent depression.

Methods: A sample of 630 adolescents (between 10 to 19 years of age) residing in the selected area of Ballabgarh (Haryana) were selected using simple random sampling technique. Home visits were made, in which participants were administered the Patient Health Questionnaire (PHQ)-9 to screen for depression. The prevalence and 95%CI were estimated. In addition, a semi-structured interview schedule was administered to identify sociodemographic variables and other factors associated with adolescent depression. Unadjusted and adjusted OR were reported with p-value, using multivariable logistic regression analysis.

Results: The age-adjusted prevalence of depression among adolescents was 20.6% (95% CI: 16.9-24.2). The prevalence in late and early adolescence was 11.7% and 8.9%, respectively. It was higher in girls (22.3%) as compared with boys (19.2%). Mild depression was the most common type identified. On multivariable logistic regression, depression was associated with birth order of four or more (Adjusted OR (AOR)=3.0 (95%CI: 1.4-6.3), p<0.01), presence of long-standing illness in the past three months (AOR=3.0 (95%CI: 1.4-6.1), p<0.01), impaired self-perceived body image (AOR=2.9 (95%CI: 1.8-4.6), p<0.01), and perceived stressful event(s) in the past six months (AOR=4.9 (95%CI: 2.8-8.6), p<0.01).

Conclusion: One in five adolescents was screened positive for depression, necessitating focus on screening and early identification of depressive symptoms, especially at the primary care level.

## Introduction

Nearly 200 million Indians were suffering from mental health disorders as per the Global Burden of Disease report (1990-2017), contributing 4.7% to total disability-adjusted life years (DALYs) [1]. Depressive disorders formed a fourth of the numbers, and a third of the DALYs was contributed by mental health disorders. Prevalence of any mental morbidity among adolescents was reported as 7.3% (95%CI: 5.8-8.7) in a pilot study from the National Mental Health Survey of India (2015-16) [2]. The growing attention on mental health disorders is not surprising, considering the rising burden of disease, disability, and deaths due to suicide [3].

The adolescent phase of life is recognized by the WHO as the age group between 10 to 19 years [4]. A fifth (253 million, 20.9%) of the Indian population is adolescent [5]. Depression is ordinarily missed in adolescents as compared to the adult population, likely due to certain dissimilar symptoms in the former that may easily be overlooked such as irritability, mood reactivity, and wavering symptoms [6]. In addition, help-seeking is less common due to fear of stigmatization [7]. Depression is associated with poor scholastic performance and unauthorized school absenteeism, low self-esteem, bullying behaviour (as victim or perpetrator), eating disorders, substance use, frequent exposure to violence, and poor sexual and reproductive health [3,8]. Depression is tied with deliberate self-harm behaviour and suicidal ideation among adolescents [3]. Early onset of depression and the persistence of depressive symptoms throughout adolescence has a lasting impression on adulthood: it affects the health of the adult (mental health disorders, substance use disorders, and non-communicable diseases such as diabetes mellitus), the health of their children, social functioning, and criminal activity [9].

Most studies conducted on depressive disorders in the adolescent age group in India are based out of school/institution-setting. A pilot community-based study was undertaken among 13- to 17-year-old individuals in the National Mental Health Survey of India (NMHS) (2015-16), and the report recommended the survey be amplified and diversified to cover a larger population of adolescents pan-India [2]. Emphasis on promotive, preventive, and curative facets of adolescent mental health has been placed in the national health programme targeted towards adolescent health, Rashtriya Kishor Swasthya Karyakram (RKSK) [5]. Against this backdrop, the current study was conducted among adolescents, investigating the magnitude of depression in a rural community, within a north Indian setting. The prevalence of depression was estimated among adolescents residing in the villages of Ballabgarh block, Faridabad district, Haryana, and factors associated with depression were identified.

## Materials and methods

Study setting

The study was undertaken from May to July, 2019, in the villages of the Intensive Field Practice Area (IFPA) under the Comprehensive Rural Health Services Project (CRHSP), Ballabgarh block, district Faridabad, Haryana, India. It caters to a population of about one lakh, residing in 28 villages. Under the CRHSP, a psychiatry outpatient clinic is held every day at the subdistrict hospital, and once a week at the primary health centre.

Study population and sampling strategy

A sampling frame of 16,709 adolescents, referring to individuals aged 10 to 19 years, was obtained from the computerized database, Health Management Information System (HMIS), maintained at the subdistrict hospital, Ballabgarh. The HMIS is regularly fed with data collected by health workers during fortnightly household visits and the annual census, thus making it a comprehensive and up-to-date database of individuals residing in the IFPA. A study conducted by Mishra, et al (2018) in Varanasi, Uttar Pradesh, was used as the reference study, according to which the prevalence of depression was 14.5% [10]. Relative precision of 20% and an alpha value of 0.05 was used to calculate the sample size. An addition of a non-response rate of 10% gave the final sample size as 630 individuals, drawn using computer-generated simple random sampling technique. Inability to comprehend the interview schedules and illness too severe to cooperate in the study were stated as exclusion criteria.

Study instruments

A thorough literature review and inputs from experts were used to design an interview schedule in a semi-structured format, which would incorporate details as perceived and reported by the participants. It included socio-demographic characteristics of the participants such as participant’s education and work-related details, information related to their communication with family members about their (participant's) day-to-day activities and whereabouts, and other personal details relevant to depression, such as history of substance use, recent stressors (which had occurred in last six months) and self-perceived body image with reference to their weight. Minimum one hour of moderate-to-vigorous physical activity carried out on a daily basis was considered adequate. Factors such as body image and physical activity were included in the interview schedule because they were found to be associated with depression during review of literature [11]. Chronic illness was defined as an illness with a duration of three months or more, or recurrent illness. The interview schedule was pre-tested and revised accordingly, and thereafter administered in local vernacular.

Patient Health Questionnaire (nine-item version) (PHQ-9) was used to screen for depression (See Appendix). PHQ-9 is a short scale consisting of nine questions, which are symptoms of depression, enabling criteria-based diagnosis of depression. The score assigned to each of these items ranges from zero (not at all) to three (nearly every day), thus allowing for a minimum and maximum score of zero and 27, respectively, with higher scores indicating more severity of depression [12]. A cut-off score of five is 87.1% sensitive and 79.7% specific for depression among Indian adolescents [13]. A diagnostic tool was used for participants who screened positive for depression using PHQ-9; its results are reported separately [14]. The single interviewer (AM), a medical graduate, was trained in the psychiatry outpatient clinic of All India Institute of Medical Science (AIIMS), New Delhi, under the supervision of an experienced psychiatrist, in the administration of study tools.

Study process

Data were collected during house-to-house visits. The interviewer was accompanied by a local volunteer to aid in identifying houses and building rapport with the participant and caregiver. If an individual was not found on day one of the visit, the next visit was scheduled after confirming their availability from family members/neighbours from the community. If not found on the next (second) visit, the individuals were listed as non-responders. The semi-structured interview schedule was administered according to the PHQ-9. Participants who scored five or more on PHQ-9 were identified as screen positive for depression. Such participants were administered a diagnostic tool, and its results are reported separately [14].

Statistical analysis

For statistical analysis, we used Stata Statistical Software: Release 12 (2011, StataCorp LP, College Station, Texas). The per cent prevalence of depression was stated with limits corresponding to 95%CI. The association of depression with factors in the interview schedule was identified using univariable and multivariable logistic regression analysis. A cut-off value of p=0.25 was used to select variables to be entered in the multivariable regression model. Unadjusted and adjusted OR and 95%CI were computed and considered statistically significant if the p-value was less than 0.05.

Ethics clearance

Ethics clearance was obtained from the Institute Ethics Committee, AIIMS, New Delhi (Ref. no. IECPG-23/23.01.2019). Each adolescent was first briefed about the purpose of the study and given a participant information sheet. Participants and their caregivers were explained about confidentiality. Informed written consent was obtained from adult participants, and adult parents/caregivers of minor participants. Written assent was also obtained from participants aged less than 18 years. In the event that a participant was identified with depression on the diagnostic tool, the situation was explained to the parent/caregiver, and consultation in the psychiatry outpatient department at the nearest health facility was recommended and referral was done.

## Results

Descriptive details of study participants

**Figure 1:**
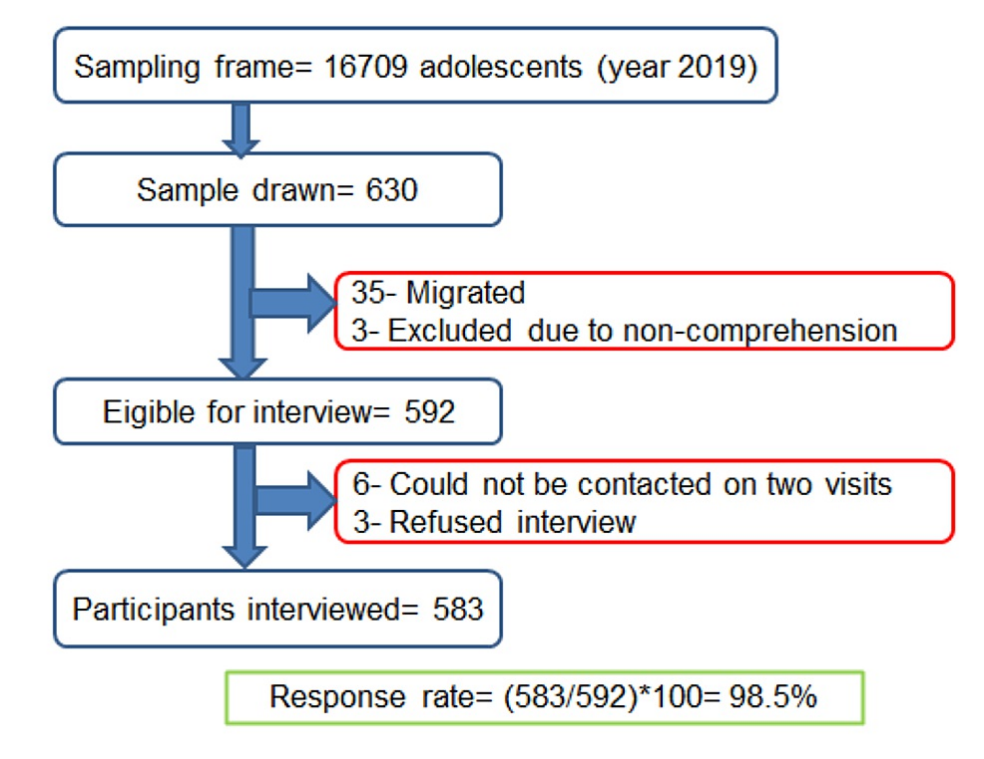
Study participant flow

Home visits were made for the selected 630 adolescents during which 35 could not be located owing to migration out of the district and three were unable to comprehend the interview schedule because of intellectual disability. These 38 participants were excluded from the study. Six adolescents could not be contacted on two occasions; the houses of two of them were locked on both visits. Three adolescents refused participation. Thus, 583 adolescents were interviewed, amounting to a 98.5% response rate. The study participant flow is described in Figure 1.

The distribution of study participants by age and sex was comparable in the study population and the sampling frame. There were 330 (56.6%) boys and 253 girls (43.4%) in this study. A total of 313 (53.7%) participants belonged to the age group 10-14 years, while 270 (46.3%) were aged 15-19 years. Three participants (one boy, two girls) were married. Eight boys and six girls, forming 2.4% of the study population, were engaged in paid work; eight, full-time, and six, for a part of the day. Thirty-seven participants had discontinued education. The percentage of study participants living with both, one, and no parents, was 92%, 7%, and 1%, respectively.

The sociodemographic characteristics of the participants have been summarized in Table 1.

**Table 1:** Sociodemographic characteristics of participants

Variable	Category	Boys n=330 (%)	Girls n=253 (%)	Total n=583 (%)	p-value (χ^2^)
Age	10-14 years	183 (55.4)	130 (51.4)	313 (53.7)	0.33
	15-19 years	147 (44.6)	123 (48.6)	270 (46.3)	
Participant’s education	Higher than 8^th^ standard	130 (39.4)	117 (46.2)	247 (42.4)	0.17
	6^th^-8^th^ standard	120 (36.4)	88 (34.8)	208 (35.7)	
	Upto 5^th^ standard	80 (24.2)	48 (19.0)	128 (22.0)	
Type of family	Extended	155 (47.0)	112 (44.3)	267 (45.8)	0.52
	Nuclear	175 (53.0)	141 (55.7)	316 (54.2)	
Marital status of parents	Married	302 (92.6)	239 (94.8)	541 (93.6)	0.28
	Others (divorced/ widowed/ separated/ both dead)	24 (7.4)	13 (5.2)	37 (6.4)	
Number of parents living with the participant	Two	299 (90.6)	238 (94.1)	537 (92.1)	0.33
	One	27 (8.2)	14 (5.5)	41 (7.0)	
	Zero	4 (1.2)	1 (0.4)	5 (0.9)	
Number of siblings	Upto two	229 (69.4)	139 (54.9)	368 (63.1)	<0.01
	Three or more	101 (30.6)	114 (45.1)	215 (36.9)	
Birth order	Upto three	273 (82.7)	220 (87.0)	493 (84.6)	0.16
	Four or more	57 (17.3)	33 (13.0)	90 (15.4)	
Father’s education	Higher than 10^th^ standard	102 (30.9)	83 (32.8)	185 (31.7)	0.86
	6^th^-10^th^ standard	171 (51.8)	128 (50.6)	299 (51.3)	
	Upto 5^th^ standard	30 (9.1)	19 (7.5)	49 (8.4)	
	Illiterate	27 (8.2)	23 (9.1)	50 (8.6)	
Mother’s education	Higher than 10^th^ standard	35 (10.6)	28 (11.1)	63 (10.8)	1.0
	6^th^-10^th^ standard	123 (37.3)	92 (36.4)	215 (36.9)	
	Upto 5^th^ standard	60 (18.2)	47 (18.6)	107 (18.4)	
	Illiterate	112 (33.9)	86 (34.0)	198 (34.0)	

Chronic illnesses were reported by 8.4% of participants. A total of 16.3% of participants cited having experienced one or more stressful events in the past six months. Nearly 60% of these participants reported reasons related to dissatisfaction with their scholastic performance or anticipation of academic results. About 30% had witnessed injury/illness to self/loved ones or death of close relatives or a loved one. Legal issues and other stressors were reported by 18% of participants. Three participants had faced more than one stressful event in the past six months.

Parents/caregivers of 57.3% (n=334) participants had used at least one substance in the past one year; 30% (n=175) participants had used two substances in the past one year, while those of 1.4% (n=8) participants had used three. The most commonly used substance was tobacco (n=290, 86.8%), followed by alcohol (n=226, 67.7%). Eight (1.4%) participants had used substances in the past one year, and seven had used substances in the past one month too. Tobacco had been used by all eight participants using substances, and alcohol by two participants.

Prevalence of depression

The prevalence of depression among adolescents using PHQ-9 was 20.9% (95%CI: 17.7-24.4) (n=122). It was higher in late adolescence (27.0% (95%CI: 21.8-32.8)), as compared with that in early adolescence (15.6% (95%CI: 11.8-20.2)).

The age-adjusted prevalence of depression was 20.6% (95%CI: 16.9-24.2). The prevalence was higher in girls (22.3%) as compared with boys (19.2%).

It was noted that with rising PHQ-9 scores, corresponding to increasing severity of depression, the number of participants identified with such severity was declining. Thence, mild depression was the most common type, identified in 72.1% (n=88) of the depressed participants, followed by moderate, moderately severe, and severe depression in 17.2% (n=21), 9.9% (n=12), and 0.8% (n=1) participants, respectively.

Association of sociodemographic and other variables with depression

On bivariable logistic regression, the odds of depression identified using PHQ-9 were significantly higher (p<0.05) among 15- to 19-year-old participants, those with lower education (primary and middle school), those with higher birth order, and those working for pay. Participants who were dissatisfied about their appearance with respect to their body weight, or were currently suffering from illness for more than three months, also had higher odds of depression, so did those perceiving their family members quarrelling/ getting into conflicts among themselves frequently, and those who had experienced one or more events in the past six months that were perceived as particularly stressful. These variables were incorporated in the multivariable logistic regression model. In addition, factors such as maternal education, number of siblings, marital status of parents, awareness of family members about how the participant spends time, family history of psychiatric illness, and substance use by the parent/caregiver in the past one year were also included for analysis in the multivariable model (0.05 ≤ p ≤ 0.25) (Table 2, Table 3). On multivariable logistic regression, depression identified using PHQ-9 was associated with birth order of four or more (Adjusted OR (AOR)=3.0 (95%CI: 1.4-6.3), p<0.01), presence of chronic illness in the past three months (AOR=3.0 (95% CI: 1.4-6.1), p<0.01), impaired self-perceived body image (AOR=2.9 (95% CI: 1.8-4.6), p<0.01), and perceived stressful event(s) in the past six months (AOR=4.9 (95% CI: 2.8-8.6), p<0.01). The association of depression with other variables did not reach statistical significance. (Table 2, Table 3)

**Table 2:** Association between sociodemographic variables and presence of depression by PHQ-9 PHQ-9: Patient Health Questionnaire-9

Variable	Category	Frequency (n=583)	Depression positive (n=122) n (%)	Unadjusted OR (95% CI)	p-value	Adjusted OR (95% CI)	p-value
Age group	10-14 years	313	49 (15.6)	1.0	-	-	-
	15-19 years	270	73 (27.0)	2.0 (1.3-3.0)	<0.01	0.9 (0.5-1.9)	0.87
Sex	Male	330	64 (19.4)	1.0	-	-	-
	Female	253	58 (22.9)	1.2 (0.8-1.8)	0.30	-	-
Participant’s education	Higher than 8^th^ standard	247	67 (27.1)	1.0	-	-	-
	6^th^-8^th^ standard	208	38 (18.3)	0.6 (0.5-5.9)	0.03	0.7 (0.4-1.3)	0.27
	Up to 5^th^ standard	128	17 (13.3)	0.4 (0.2-0.7)	<0.01	0.6 (0.2-1.4)	0.20
Father’s education	Higher than 10^th^ standard	185	37 (20.0)	1.0	-	-	-
	6^th^-10^th^ standard	299	64 (21.4)	1.1 (0.7-1.7)	0.71	-	-
	Upto 5^th^ standard	49	8 (16.3)	0.8 (0.3-1.8)	0.56	-	-
	Illiterate	50	13 (26.0)	1.4 (0.7-2.9)	0.36	-	-
Mother’s education	Higher than 10^th^ standard	63	10 (15.9)	1.0	-	-	-
	6^th^-10^th^ standard	215	51 (23.7)	1.6 (0.8-3.5)	0.19	0.9 (0.4-2.1)	0.86
	Up to 5^th^ standard	107	22 (20.6)	1.4 (0.6-3.1)	0.45	0.7 (0.3-1.7)	0.40
	Illiterate	198	39 (19.7)	1.3 (0.6-2.8)	0.50	0.6 (0.2-1.6)	0.33
Type of family	Extended	267	56 (45.9)	1.0	-	-	-
	Nuclear	316	66 (20.9)	1.0 (0.7-1.5)	0.98	-	-
Number of siblings	Upto two	368	71 (19.3)	1.0	-	-	-
	Three or more	215	51 (23.7)	1.3 (0.9-2.0)	0.20	0.7 (0.4-1.3)	0.29
Birth order	Upto three	493	74 (20.0)	1.0	-	-	-
	Four or more	90	48 (22.5)	1.8 (1.1-3.0)	0.02	3.0 (1.4-6.3)	<0.01
Marital status of parents (n=578)	Married	541	109 (20.2)	1.0	-	-	-
	Others (widowed/divorced/separated)	37	12 (32.4)	1.9 (0.9-3.9)	0.08	1.8 (0.8-4.3)	0.18
Number of parents living with the participant	Zero	5	1 (20.0)	1.0	-	-	-
	One	41	14 (34.2)	2.1 (0.2-20.4)	0.53	-	-
	Two	537	107 (19.9)	1.0 (0.1-9.0)	1.00	-	-
Marital status of participant	Married	3	1 (33.3)	1.0	-	-	-
	Unmarried	580	121 (20.9)	0.5 (0.0-5.9)	0.60	-	-
Working for pay	No	569	114 (20.0)	1.0	-	-	-
	Yes	14	8 (57.1)	5.3 (1.8-15.6)	<0.01	3.6 (1.0-13.7)	0.06

**Table 3:** Association between miscellaneous variables and presence of depression by PHQ-9 PHQ-9: Patient Health Questionnaire-9

Variable	Category	Frequency (n=583)	Depression positive (n=122) n (%)	Unadjusted OR (95% CI)	p-value	Adjusted OR (95% CI)	p-value
Knowledge of family members of how the participant spends time	No	21	6 (28.6)	1.0	-	-	-
	Occasionally	72	9 (7.4)	0.4 (0.1-1.2)	0.09	0.5 (0.1-2.2)	0.38
	Yes, usually	490	107 (21.8)	0.7 (0.3-1.8)	0.47	1.3 (0.4-244)	0.63
Knowledge of family members about whom the participant spends time with	No	21	4 (23.5)	1.0	-	-	-
	Occasionally	82	18 (22.0)	0.9 (0.3-3.2)	0.89	-	-
	Yes, usually	484	100 (20.7)	0.8 (0.3-2.6)	0.78	-	-
Perceived atmosphere at home	Calm	34	14 (41.2)	1.0	-	-	-
	Occasional conflicts	191	45 (23.6)	1.4 (0.9-2.2)	0.01	1.1 (0.7-1.8)	0.69
	Frequent conflicts	358	63 (17.6)	3.3 (1.6-6.8)	<0.01	2.1 (0.9-5.0)	0.10
Family history of psychiatric illness	No	557	114 (20.5)	1.0	-	-	-
	Yes	26	8 (30.8)	1.7 (0.7-4.1)	0.21	1.2 (0.4-3.5)	0.72
Body image	About right	371	52 (14.0)	1.0	-	-	-
	Others (thin/fat)	212	70 (33.0)	3.0 (2.0-4.6)	<0.01	2.9 (1.8-4.6)	<0.01
Stressful event	No	488	75 (15.4)	1.0	-	-	-
	Yes	95	47 (49.5)	5.4 (3.4-8.6)	<0.01	4.9 (2.8-8.6)	<0.01
Chronic illness	No	534	101 (18.9)	1.0	-	-	-
	Yes	49	21 (42.9)	3.2 (1.8-5.9)	<0.01	3.0 (1.4-6.1)	<0.01
Adequate physical activity per week	Yes	445	91 (20.4)	1.0	-	-	-
	No	138	31 (22.5)	1.1 (0.7-1.8)	0.61	-	-
Substance use by parent/ caregiver in past one year	No	249	46 (18.5)	1.0	-	-	-
	Yes	334	76 (22.8)	1.3 (0.9-2.0)	0.21	1.4 (0.8-2.2)	0.20
Substance use by participant in past one year	No	575	119 (20.7)	1.0	-	-	-
	Yes	8	3 (37.5)	2.3 (0.5-9.8)	0.26	-	-

## Discussion

The age-adjusted prevalence of adolescent depression using PHQ-9 was found to be 20.6% (95%CI: 16.9-24.2). Studies conducted in the Indian community reported prevalence of depression varying from 0.5% to 16.2% [2,10,15,16]. This diversity could be explained by dissimilar sampling techniques, characteristics of the study population and region, the multitude of study instruments used, diagnostic criteria, number of stages for diagnosis (single or multiple), and training status/qualification of interviewers. Simple random sampling technique was used in this study to ascertain the representativeness of the sample with respect to the sampling frame. The study instrument, PHQ-9, is sensitive and specific for identifying depression, its brevity allowing for its use as a clinical and research tool, and it has been validated in India among the adolescent population and in Hindi [13,17]. Chauhan et al. reported the prevalence of depression among students in Noida, Uttar Pradesh, using PHQ-9, as 37.8% [18]. Singh et al. also used it as a single-stage diagnostic instrument in school-going adolescents in Chandigarh and reported the prevalence of depressive disorders as 40%, and that of major depressive disorders (MDD) as 7.6% [19]. Inconsistent cut-off scores and revisions of the study instrument may also account for the variation in the observed prevalence of depression across studies. In addition, revision of diagnostic criteria under the International Classification of Diseases (ICD) [20] and Diagnostic and Statistical Manual of Mental Disorders (DSM)-5 [21] should be taken into consideration as a contributor to the temporal change in prevalence over the years. Most studies had multiple interviewers, raising the possibility of inter-observer variability. This is ruled out in the present study since data was collected by a trained physician as the single interviewer.

Oderinde et al. administered PHQ-9 modified for adolescents (PHQ-A) to 540 students in rural Nigeria as a screening tool for depression, followed by the depressive disorders module of Kiddies-Schedule for Affective disorders and Schizophrenia (K-SADS), and reported the weighted one-month prevalence of depression as 16.3% [22]. Alharbi et al., in a recent study in Saudi Arabia [23] among 1245 students, reported a whopping 74.0% prevalence of depression using PHQ-9 as the study tool. They reported the prevalence of mild, moderate, moderately severe, and severe depression in the study population as 34%, 24.6%, 10.4%, and 5%, respectively, while it was 15.1%, 3.6%, 2.0%, and 0.2%, respectively, in our study. Thus, the prevalence of the more severe types of depression was lower than the milder types in both studies.

Experience of one or more events in the past six months, which were perceived as particularly stressful, was significantly associated with depression in this study. It is noteworthy that the number of such events, and not merely their one-time occurrence, has been associated with depression in studies [24,25]. Dissatisfaction with one’s academic performance has been widely studied as a factor associated with adolescent depression in the country and elsewhere [3,16,19,24-27]. It may be explained by school absenteeism, poor concentration in studies, lethargy and easy-fatiguability, poor self-esteem, and sleep disturbances. A factor peculiar to the Indian education system - studying in ‘board’ classes - was discovered to be associated with depression by Bhasin et al. [25]. Board exams are state and national-level examinations conducted at the end of the 10th and 12th standards and are considered important in India for securing higher education in desired institutes. Other stressful factors mentioned by the participants, related to death/illness/injury in family/to self, were also identified as stressors in other studies conducted in India and worldwide [11,24-26,28,29].

Participants who were dissatisfied about their physical appearance with respect to their body weight had higher odds of depression (AOR=2.9 (95%CI: 1.8-4.6), p<0.01). These findings have also been noted by Dooley et al. [11]. Unreasonable and unmet expectations towards one’s own appearance may lower self-esteem, bring about withdrawal from social interaction, and trigger unhealthy eating patterns linked to anorexia or bulimia, all of which are associated with depression.

The presence of chronic illness was significantly associated with depression (AOR=3.0 (95%CI: 1.4-6.1), p<0.01) on multivariable analysis. Such associations were also identified in other community-based global studies by Belair et al. and Farbstein et al. [29,30]. Farbstein et al. identified an association of internalizing disorders with congenital disorders, asthma, headaches/migraine, allergies, epilepsy, Crohn’s disease, and expert-diagnosed learning disability [30]. Shah et al. also identified the association of depression with a history of illness requiring pharmacological management in a school-based study in the UAE [28].

Higher birth order was associated with depression in this study (AOR=3.0 (95%CI: 1.4-6.3), p<0.01). Easey et al. (2019) analyzed data from Avon Longitudinal Study of Parents and Children (ALSPAC) (n=2571) to determine associations between birth order and psychiatric disorders at age 15 years identified using Development and Well-Being Assessment (DAWBA), as well as suicide attempts. Graded linear association was noted between birth order and odds of suicide attempt (AOR= 1.42 (95%CI = 1.10-1.84)), with attempts increasing with each unit of increase in birth order: for second-born (OR = 1.56, 95% CI = 1.05-2.31) and third plus born children (OR = 1.97, 95%CI = 1.17-3.34). The association of psychiatric disorders in adolescence with birth order did not reach statistical significance (p=0.06) [31].

This study is among the limited number of community-based studies on adolescent depression in India and the even fewer studies based in North India. The methodology was robust. Representativeness of the sample was ascertained by drawing an adequate simple random sample. In addition, the response rate was high (98.5%). The study instrument, PHQ-9, has been validated in adolescents and has good sensitivity and specificity, and has been used in the community setting in this study [13]. A trained medical graduate as the single interviewer assured quality data and ruled out the possibility of inter-observer bias. The mentioned factors suggest good internal validity of the study and the results may be generalized in a similar setting, i.e., the rural Indian community of this region. This is of importance, considering that most studies on depression among adolescents have been conducted in institution-based settings, which restricts extrapolation of their results to the general population. However, there is a need for the generation of evidence from multisite and multiethnic studies across the Indian landscape in order to gain an in-depth understanding of the variation in the prevalence of depression and factors responsible for the differences across regions.

We note an air of stigma around talking about mental health disorders and related questions, and the possible reluctance/hesitance of the study participants in disclosing the true state of their mental health, surrounded by their kins. In order to ensure privacy, the participants were interviewed either in their home or in a secured community space where there were no interruptions, with the consent of the parents/caregivers and communication about the purpose of the study and confidentiality of data. Rapport was established with the help of a local volunteer to ensure the validity of the data. Questions about intrafamilial conflicts and discord, and substance use by self/caregiver could have invited responses reflecting social desirability bias. Single-stage diagnosis could lead to overestimation of point prevalence, as discussed in a review by Grover et al. [32]. Further assessment using a diagnostic tool is warranted, the results of which are reported separately [14]. Since the study design was cross-sectional, the causal association could not be determined. As an extension, bidirectionality could not be ruled out for certain variables, such as physical activity and substance use and their association with depression. Also, the variables studied as factors associated with depression may be explored further with a larger sample size, which may give more precise results.

## Conclusions

One in five adolescents was screened positive for depression, necessitating focus on screening and early identification of depressive symptoms, especially at the primary care level. This could limit the functional impairment due to depression and improve scholastic performance, the deterioration of which was identified as a stressor in numerous study participants. Since depression in adolescence is prone to recurrence and is a predictor of psychiatric and medical illnesses in adulthood, early management could pave the way to a better future for affected adolescents. Training medical undergraduate students and primary care physicians to identify common mental disorders and orienting post-graduate psychiatry students to child and adolescent psychiatry, complemented with increasing the superspecialty vacancy for education in this discipline, could address the existing scarcity of skilled mental health professionals in this domain and reduce the treatment gap. With the expansion of comprehensive primary healthcare services in rural India and the rolling out of mental healthcare package within it, the primary health workforce should be oriented well in picking up and adequately managing common mental disorders, such as depression.

Taking into account the association of stressful events with depression, a low-stress lifestyle should be encouraged. Adolescents with chronic illnesses should be screened for depression and offered appropriate mental health services to limit functional impairment.

## Figures and Tables

**Figure 2:**
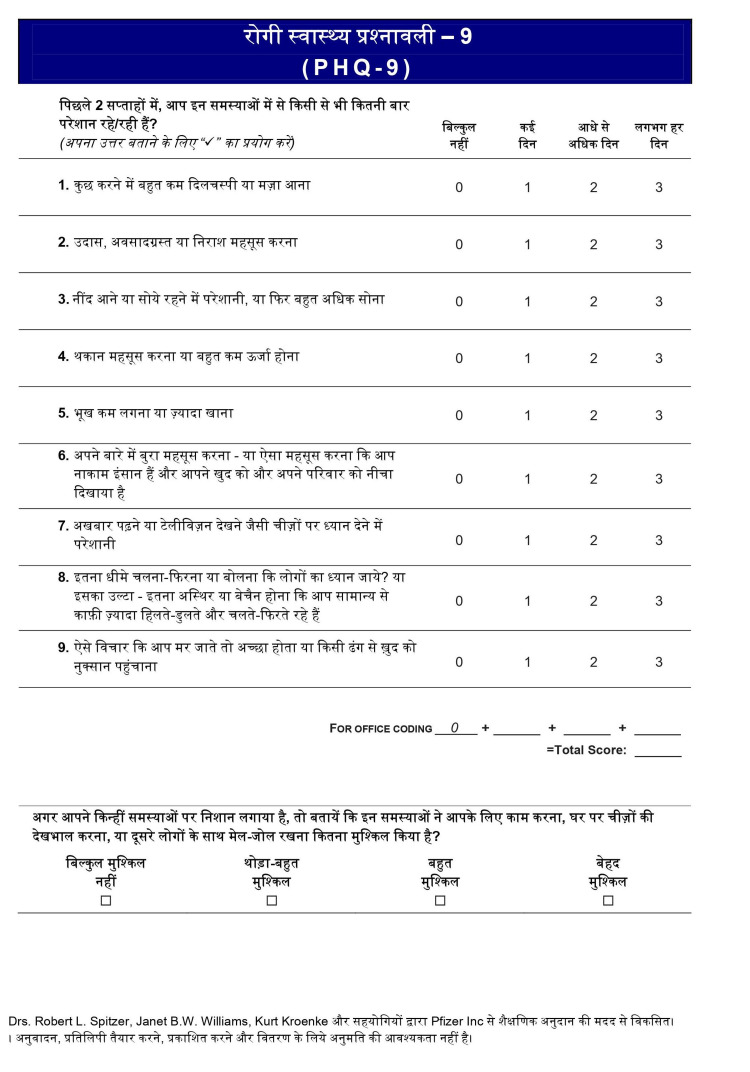
Patient Health Questionnaire-9 in Hindi used in the study
